# Women’s involvement and engagement in climate and health research in two Global South countries - Indonesia and Malaysia: barriers and facilitators

**DOI:** 10.1186/s40900-026-00953-x

**Published:** 2026-09-21

**Authors:** Raksha Pandya-Wood, Gabriela Fernando, Savyna Selvarajan, Dessy Rosalina, Azliyana Azhari, Nadira Chairani, Grace Wangge

**Affiliations:** 1 Translational Health Research Institute, Western Sydney University, Sydney, Australia; 2 Public Health, Monash University Indonesia, Bumi Surpong Damai, Indonesia; 3 Monash Climate Communication Hub, Monash University Indonesia, Bumi Surpong Damai, Indonesia; 4 Monash Climate Communication Hub, Monash University Malaysia, Bandar Sunway, Selangor, Malaysia

**Keywords:** Women, Climate change, Global south, Health, Research, Involvement, Southeast Asia, Partnership

## Abstract

Climate change disproportionately affects women in low- and middle-income countries (LMICs), where entrenched gender roles shape vulnerability and response capacities. Many women are often responsible for post-disaster clean-up, caregiving for children and relatives affected by climate-related health impacts, and ensuring household food and water security during periods of scarcity. These roles place many women at the frontline of climate-related hardship, yet their perspectives remain underrepresented in climate and health research. Addressing this gap is critical for generating evidence that is both equitable and grounded in the realities of those most affected. This study sought to explore conditions under which a snapshot of women in Southeast Asia could move beyond participation toward partnership in future research and to examine pathways for their active involvement in research, not merely as participants but as partners in shaping agendas, methodologies, and outcomes. We conducted workshops in Indonesia and Malaysia to examine women’s potential roles across the research cycle, their training needs, and the barriers and enablers to involvement and engagement in research. Discussions revealed that they are willing and able to contribute at all stages of research, provided safe, supportive spaces are created. These spaces were described as women-only environments that allowed children to be present, enabling participation without compromising caregiving responsibilities. However, a critical barrier was the requirement for men’s permission to engage in research activities, highlighting the need to negotiate access and build trust within households and communities. Religious and cultural contexts further shape these dynamics, underscoring the need for researchers and policymakers to collaborate with local institutions to strengthen community ties and facilitate women’s inclusion. Our findings indicate a snapshot of what facilitates women’s involvement as active partners in climate and health research, thereby enhancing the relevance, legitimacy, and sustainability of such research. By addressing structural barriers and embedding culturally sensitive approaches, participatory research can empower women, amplify community resilience, and generate adaptation strategies that benefit entire families and communities. The implications extend beyond Southeast Asia, offering lessons for global climate-health research on how inclusive methodologies can contribute to more equitable and sustainable responses to climate change.

**Supplementary Information:**

The online version contains supplementary material available at https://doi.org/10.1186/s40900-026-00953-x.

## Abstract (fr)

Climate change affects everyone, but in low- and middle-income countries it often affects women more than men. This is because traditional gender roles mean most women are usually responsible for cleaning up after floods, caring for sick children or relatives, and preparing meals, even when food and water are scarce. These responsibilities place extra burdens on women during climate-related disasters. We wanted to better understand women’s lived experiences in these circumstances so they could be more actively involved in shaping climate and health research. By including women as partners, not just participants, research can ask more relevant questions and lead to practical recommendations that benefit families and communities. To explore this, we held workshops in Indonesia and Malaysia. We asked 42 women across Jakarta and Kuala Lumpur how they might take part in different stages of research, what training they would need, what challenges they face, and what support could help them participate actively. These women told us they could contribute across the entire research cycle, from planning to sharing results, if safe spaces were provided. These spaces needed to be women-only and to allow children to be present. However, many of our participants explained that they often require men’s permission to take part, making negotiation of access through men an important step. Because gender roles in Indonesia and Malaysia are strongly shaped by religious norms, enabling women’s involvement requires collaboration with trusted religious and community institutions. When women participate as research partners, the findings become more grounded in local realities and strengthen community resilience to climate change.

## Background

### Contextualising climate vulnerability in Southeast Asia

Southeast Asia is among the world’s most climate-vulnerable regions, experiencing unpredictable and severe disasters such as earthquakes, floods, droughts, tsunamis, volcanic eruptions, typhoons, and cyclones [12]. The region’s high proportion of remote and rural populations, agrarian and fishing communities, and substantial Indigenous groups further heightens vulnerability, with women and children disproportionately affected.

According to UNICEF [13], women and children are 14 times more likely than men to die during a disaster, and research from Indonesia, the Lao People’s Democratic Republic (PDR), and the Philippines shows that girls and women face heightened post-disaster risks, including gender-based violence and early marriage [14]. Despite this high vulnerability, there remains limited documentation and understanding of community perspectives, particularly of those disproportionately affected, including Indigenous and marginalised women and their communities. This is especially true regarding their participation, leadership, and decision-making in responding to climate risks, in climate action, and in building climate resilience [15].

This lack of contextualised and gendered perspectives, despite clear evidence of women’s heightened climate vulnerability [14] and their leadership in adaptation and community resilience [16], is especially concerning in Southeast Asia, where policymakers have yet to allocate dedicated resources to support women’s active involvement. Gan et al. [16] highlighted how women-led grassroots networks and movements across the Asia-Pacific have been pivotal in climate action and health resilience. They also note that women’s voices remain under-represented in decision- and policy-making across Southeast Asia, despite women’s central roles in caring for family during disasters, in agrifood systems, in water management, and in disaster responses. Research also shows that among Southeast Asia’s Indigenous peoples, inclusion in adaptation and health-preparedness planning is unequal, with many communities facing increasing physical and mental health risks, displacement, and high food insecurity due to land degradation, biodiversity loss, and climate stressors [17]. Despite this, Indigenous perspectives continue to be frequently neglected and marginalised. Power structures entrenched in colonial legacies and limited institutional recognition undermine Indigenous voices, particularly Indigenous women’s contributions to climate justice and action [17].

Climate and health research in low and middle income countries (LMICs) has focused on how climate variability affects health through direct impacts such as heat stress, vector-borne diseases, and extreme weather-related injuries, and through indirect pathways such as food insecurity, displacement, livelihood disruption, and weakened health systems [7]. These impacts are deeply gendered: women and girls often face higher exposure to climate-related risks due to unequal access to resources, caregiving burdens, mobility restrictions, and occupational segregation [8], as well as limited health decision-making agency [9].

Bringing Indigenous women’s voices to the fore requires research grounded community participatory and inclusive methodologies. These approaches must centre the lived experiences of marginalised and vulnerable women, including Indigenous women, and explicitly acknowledge the intersecting identities, needs, and inequalities that shape these populations’ climate and health vulnerabilities. To address this, using participatory and qualitative research approaches, this study explores how a small group of women from two countries (Indonesia and Malaysia) can engage meaningfully in research processes on the climate-health nexus. The contribution of this work lies in providing nuanced empirical insights into how to involve and centre women’s voices and leadership, and into the barriers and enablers shaping women’s involvement across diverse social and institutional contexts.

### Situating public involvement in this research

Patient and Public Involvement (PPI) in research refers to the active involvement of patients, service users, carers and community members as partners in research, who contribute to shaping research priorities, design, conduct, interpretation, and dissemination, rather than being treated solely as research participants or data sources. INVOLVE developed in the UK context, defines PPI as research undertaken *with* or *by* members of the public, not *to*, *about*, or *for* them [1], and positions co-design and co-production as central to meaningful involvement [1–3].

While INVOLVE’s definition informed the aspirations and conceptual grounding of the present study, full realisation of this was not achievable within the study context. In Indonesia and Malaysia, where our study was conducted, there are no established or funded infrastructures to support early-stage public involvement in climate and health research and policy-related decision making, which limits opportunities for agenda-setting or co-design at the point of study conception.

Our focus therefore was to address the barriers to and facilitators of women’s (including Indigenous women’s) active involvement and engagement in climate and health research. Set in Indonesia and Malaysia, both deeply patriarchal and religious countries and systems, our research sought to explore the capacities, processes and factors influencing PPI in research. Evidence from scoping [4] and systematic reviews [5] shows that communities and researchers in LMICs face systemic barriers. LMICs are constrained by limited local funding, power imbalances, missing infrastructure, and a lack of guidance from funders [6].

Essex et al. [10] argue that traditional semicolonial approaches to research often exploit communities in LMICs. They call for sustainable PPI practice in research that empowers communities, enhances legitimacy, and ensures that the research benefits the communities who are the likely beneficiaries. Cook et al. [5] reinforce this by showing that PPI in LMICs, although inconsistently reported, can improve the relevance and appropriateness of research and strengthen trust between researchers and community members. In climate and health research, this principle can be extended to integrate communities in research-related planning, conduct, implementation, and beyond. Most notably in recent times, this principle is evident in the way the Loss and Damage Fund is operationalised through community-led adaptation efforts [11] when a climate disaster occurs.

Conceptually, this study also draws on Arnstein [1] ladder of citizen participation, which frames participation as a matter of power and decision‑making authority rather than merely the presence of engagement activities. Arnstein distinguishes between tokenistic forms of participation, such as informing and consultation, and higher levels of citizen power, including partnership and community control. Using this framework as an analytic heuristic, rather than a normative benchmark, this study was positioned primarily within consultative and early partnership‑oriented forms of involvement. Through facilitated workshops, the research created structured spaces for dialogue, advice-giving, priority reflection, and partial involvement in later stages of the research cycle, while recognising that movement towards community-led research requires longer-term investment, institutional support, and governance structures that extend beyond the scope of this project.

When applied through a gendered lens in LMICs, PPI becomes a mechanism for equitable and transformative climate-health research centred on women’s voices, recognising gender-specific vulnerabilities and capacities, addressing power asymmetries, and enabling the co-production of policies and interventions that reflect women’s needs and priorities.

While women’s voices were actively sought, valued, and influential within facilitated research spaces, decision‑making authority over research design and governance largely remained with the academic team. This positioning reflects not a lack of interest or capacity among women, but the structural, cultural, and institutional constraints shaping involvement in climate and health research more generally in low‑ and middle‑income (LMIC) country contexts. While much formalised PPI guidance originates from high‑income country contexts, important models of community engagement have also emerged from well‑resourced LMIC research centres, particularly those supported through long‑term external funding. However, such models are often locally concentrated and difficult to replicate more broadly in the absence of sustained institutional infrastructure, policy mandates, and resourcing. The current study therefore rests in the consultative and exploratory involvement category, not co-production.

## Methods

### Aim

The study explores how to create more inclusive research practices with women in Indonesia and Malaysia to move beyond participation in climate and health research and towards forming partnerships and shaping climate research agendas. Within this we will explore roles in the research cycle; how intersectional identity shapes their involvement; approaches to engagement and barriers and facilitators to creating more involvement in research opportunities.

The study adopted an interpretivist approach. Interpretivism holds that social reality is socially constructed, and therefore researchers must interpret the lived experiences, meanings, and perspectives of individuals or groups [18]. The design integrated qualitative approaches through a series of participatory workshops.

### Ethics

Due to the study’s multi-country nature and funding requirements, ethical approval was required and obtained from three organisations: Monash University (ID:45509), the National Research and Innovation Agency Indonesia (Badan Riset dan Inovasi-BRIN, No: 084/KE.01/SK/02/2025), and the World Health Organisation (ID: ERC.0004217).

This study was guided by established ethical principles, including respect for autonomy, non-maleficence, beneficence, and relational ethics, with particular attention to culturally situated and gender-sensitive research practice. All participants were provided with study details in their preferred language to ensure informed consent [19]. Recognising that consent is ongoing and context-dependent, participants were reminded of their rights to withdraw at any stage or to refuse to answer any questions they were uncomfortable with, without consequences. To ensure participants’ wellbeing, multiple safeguarding measures were implemented. Workshops were designed to create safe, supportive spaces for participation, to respect cultural and religious norms, and to provide opportunities for participants to share feedback and raise concerns. Local researchers in our teams were trained in neutrality, facilitation, and integrity. The workshops were conducted in local languages to ensure accessibility (Ibid). Bahasa Indonesia was used in Indonesia, and English and Bahasa Melayu were used in Malaysia. Facilitators were briefed to manage sensitive discussions, recognise distress, and respond appropriately to participants’ needs, including referral pathways where necessary. Cultural sensitivities were addressed by grouping participants according to religious and ethnic backgrounds [20]. Prayer facilities were provided, and safe women-only spaces were created, allowing children to accompany their mothers if needed.

To ensure confidentiality and data protection, all data were anonymised at the point of transcription, with identifying details removed or coded. Data were securely stored on password-protected institutional servers, accessible only to the research team, in accordance with institutional and international data protection guidelines. Given the potential sensitivity of participants’ experiences, particular care was taken to minimise the risk of identification, especially for individuals from vulnerable or marginalised groups.

### Recruitment and sample selection

The study population comprised women aged 18 years and above residing in the greater Jakarta, Indonesia (*n* = 24), and Kuala Lumpur, Malaysia (*n* = 18). The larger Indonesian sample reflected the country’s greater geographical diversity, higher poverty rates [14], and a more complex climate-health profile [17].

Women ranged in age from 18 to 65 (see Appendix 3). Participants included both community members with no prior involvement in research and women working professionally in research, policy, or civil society organisations, whose perspectives offered insight into different but interrelated dimensions of involvement and engagement. They reflected Indonesian ethnicities, including Javanese, Sundanese, Madurese, Minangkabau, Batak, Bugis, Balinese, Acehnese, Dayak, and Chinese Indonesian. In Malaysia, the women were Malay Bumiputra, Chinese, Indian, and Orang Asli. Religions included Muslim, Christian, Buddhist, and Hindu, alongside diverse nature-worshipping customs among Indigenous groups. Participants spanned the full continuum of marital status, including unmarried, cohabiting, partnered, separated, and married individuals. Women in the sample experienced a range of health challenges linked to climate-related impacts, including heat-related illnesses, respiratory and infectious diseases, malnutrition, mental health stressors, and pregnancy- or caregiving-related complications. Whilst these conditions were not exclusively tied to climate change, the sample included women who had experienced dengue fever, respiratory illness, heat stress, and food insecurity. Recruitment prioritised diversity across social categories to capture intersectional experiences, including disabilities and Indigenous groups. Men, children under 18, and women residing outside Indonesia or Malaysia were excluded.

To facilitate public involvement within the constraints of the study setting, an Indonesian community researcher NRC, who had experience in community-based gender and sexuality research and was not employed in a conventional academic research role at project inception, but they contributed to recruitment, workshop facilitation, interpretation of findings, and manuscript development. This involvement provided a mechanism for incorporating community-informed perspectives into the conduct and interpretation of the study.

SS and RPW in Malaysia and NCR and DR in Indonesia recruited women facilitated through partnerships with Government and community organisations working at the intersection of climate and health. Snowball sampling [21] was used to reach additional participants. All organisations were offered a one-on-one Zoom meeting prior to the research to explain the purpose of the research and what we hoped to achieve. Several other retention strategies were adopted. For example, workshops were scheduled within the same week to maintain continuity. Travel expenses were reimbursed, and hybrid participation options were offered for those unable to attend in person. Language translators and trusted community mediators, particularly for Indigenous women, were sought. Women were encouraged to discuss participation and engagement in this work with family members. Where necessary, next-of-kin consent was accepted. Written consent was collected prior to the discussion at the workshops. Verbal or text-based consent was accepted beforehand [22].

### Data collection

Malaysian workshops were held in our university classroom and Indonesian workshops were held in a community centre in central Jakarta. Both sites were equipped with Wi-Fi, whiteboards, flipcharts, and audio-visual recording facilities. Refreshments were provided. Each workshop followed a structured set of activities. The workshops should be understood as primarily consultative, with an intentional educational component to support informed discussion. While these activities enabled meaningful dialogue and advice-giving, they did not constitute full co-design of the research.

Participants were introduced to climate-health impacts in Indonesia and Malaysia. Involvement and engagement in research, an entirely new concept to most of them, was explained using simplified research terms and examples from high-income countries and other middle-income countries, where available. In Malaysia we invited an academic speaker to share an example of how they had conducted PPI in their study. In Indonesia NCR, DR AND GW carried out this demystification process. This part of the workshop was aimed at building research literacy. Sharing sessions were held in which women previously involved in research as co-researchers shared their experiences in local languages. Photographs were used to demystify what involvement and engagement in the research process looked like. After this, group discussions explored barriers and facilitators to involvement. Brainstorming at each table identified roles women could play across the research cycle. Discussions also examined examples of research, involvement and engagement from other climate-vulnerable low- and middle-income countries. Data was collected using multiple methods. Audio recordings captured table discussions. Photographs documented workshop materials such as post-it notes and flipcharts. Facilitators kept detailed field notes.

### Reflections from our fieldnotes on data collection

Our team of researchers at the time all resided in Indonesia and Malaysia and maintained strong links to local communities. Our work is shaped by long-standing lived engagement with the social, linguistic, cultural, and environmental contexts - even though all of us are academic researchers, except for NRC. NRC was community-based at project inception but later temporarily affiliated through project funding - at the workshops their community knowledge was principally drawn upon. The researchers remained critically attentive to power relations and Global North–Global South knowledge hierarchies. As a result, the intentional choice to gather insights through researchers and community members facilitated workshops, enhanced collaboration and engagement with participants, creating space for inclusive, open, and transparent dialogue to share experiences, priorities, and collective insights. We accept that community-led data collection may sit at the boundary between participation and involvement.

### Data analysis

Data analysis followed a thematic analysis approach as outlined by Braun and Clarke [23]. This method was well-suited to interpretivist research and enabled exploration of patterns across qualitative data while remaining grounded in participants’ lived experiences.

Analysis proceeded through several stages. First, bilingual researchers (GW, DR and NCR in Indonesia, and AA and SS in Malaysia) familiarised themselves with the data. Audio recordings were transcribed verbatim and translated into English. Transcripts, notes, and visual materials were reviewed to gain an overview of the data. Second, initial codes were generated inductively to capture meaningful segments of data relating to barriers, facilitators, and potential roles. Coding was conducted by multiple researchers from the team (RPW, SS and GF in Malaysia and DR, NCR and GF in Indonesia) to enhance reliability. In this step, we were also guided by Lincoln and Guba’s [24] trustworthiness criteria (credibility, transferability, dependability and confirmability). Third, codes were collated into potential themes that reflected broader patterns across the dataset. Themes were reviewed and refined to ensure coherence and distinctiveness. Fourth, final themes were defined and named. Illustrative quotes from participants were used to support each theme. Finally, the reported themes highlight both country-specific and cross-country findings. The rich contextual notes from the facilitators, weekly team meetings during analysis, and reflexive triangulation helped to add rigour and ground the resulting themes.

## Results

The themes we share here are *Roles in the research cycle*,* Gender and caregiving*,* Approaches to engagement*,* and Trust*,* collaboration and leadership.* These themes are presented because they most directly illuminate barriers to and enablers of women’s involvement and engagement in climate and health research, which is the analytic focus of this article. Taken together they illustrate a rich and complex knowledge base advancing understanding of women’s roles in climate-health research and also point to practical pathways for building trust, strengthening collaboration, and embedding intersectional perspectives in climate and health research in similar LMIC settings.

### Roles in the research cycle

Reflections within this theme drew on both women’s perspectives as members of the public and, for some participants, their professional experience in research or policy settings. While distinct, these viewpoints were analytically valuable in illustrating how gendered barriers operate across community engagement and institutional research contexts.

Women in both Indonesia and Malaysia articulated clear roles they could contribute to across the research cycle. In planning and design, women expressed confidence in shaping research questions, validating methodologies, and co-defining objectives to ensure relevance and feasibility. For example, IP14, a researcher at a government institution who can support this stage, said:

I can formulate questions… researching the problem, reviewing the literature, mapping it accordingly… even reaching out to policymakers if necessary. (IP14, Indonesia)

And MP5, who serves as a bridge between communities and institutions, using her platform to amplify grassroots voices, said:

We will also have to check and validate their methodology, their justification, whether it sounds concrete. (MP5, Malaysia)

In the data collection phase, participants emphasised their networks and lived experiences as assets. For some women who had not considered research involvement before, their only point of reference for female researchers was that a significant portion of national Census enumerators were women [26]. IP9, an employee at an Indonesian institution that empowers women-headed households, stated that women were seen as more ‘usually more diligent’ and trusted by their communities.

According to Investing in Women [27], women data collectors can help foster trust and rapport, increasing the likelihood of full cooperation and candid responses, particularly in diverse and stratified societies where gender dynamics shape household privacy and the disclosure of sensitive topics such as sexual and reproductive health and domestic issues.

In data analysis, women described their future roles in correcting misinformation and ensuring cultural accuracy, such as clarifying misinterpretations of traditional practices. MP3, an Orang Asli woman who can offer lived and accurate experiences of other Orang Asli women, said that she is highly capable of correcting researchers on traditional norms affecting her culture:

I can then help correct any misinformation about the Sewang [dance]. I can help when the researchers get information wrong. (MP3, Malaysia)

In line with the earlier comment about diligence, women also agreed that they were more careful reviewers of findings than men. IP9, who is eager to contribute to rural women’s research initiatives, said:

Women are more meticulous than men, you know. Well, their persistence is also related to that sensitivity. (IP9, Indonesia)

Finally, under this broad theme of involvement across the research cycle, the women shared their views on their willingness to draft guidelines, mobilise community-based funding, and lead dissemination through policy briefs, popular communication, and even influencer roles.

In the funding space, IP13, who works at a women’s health NGO and has a public health background, argued that it is ethical to secure funding to train communities in aspects of the research process, such as writing research proposals:

So funding must also be able to cover not only research costs, but also how people can [get involved in] … training in writing proposals for funding. (IP13, Indonesia)

Although there is very little documented information on the training needed by the public interested in conducting research in LMICs, this statement implies that women want to learn and actively contribute to research, and that funding is critical to support this. More broadly, participatory research and adaptation projects in Asia-Pacific, Africa, and Latin America demonstrate that, when supported with resources, women from local communities have helped shape ethical frameworks (e.g., ensuring community consent, cultural sensitivity, and benefit-sharing in data collection and intervention design) in climate and health research [16].

Regarding mobilising funding, women described faith-based women’s organisations, including those with a religious studies or charitable orientation, as increasingly recognised as influential funders and advocates for climate resilience and health research at both community and transnational levels [28]. In Indonesia, one of our participants (IP8), who works at a faith-based NGO, stated that:

In my NGO, I also often raise funds through crowdsourcing, much of which comes from women’s religious studies or charity. (IP8, Indonesia)

Some participants spoke about helping to disseminate research to the community through workshops or training. For example, MP13, who has conducted capacity-building activities with migrant workers, used research-informed training to build understanding of labour rights.

In the growing literature from LMICs, it has been documented that women in vulnerable communities have led workshops and capacity-building sessions [29]. These forums empower women not only as knowledge recipients but also as knowledge producers and disseminators within their communities. Considering dissemination and what comes with the knowledge-sharing phase, Lassi and Salam’s [30] work, not specific to LMICs, has documented how women have been actively involved in translating research insights into policy dialogues and community action plans. Women discussed how social influencers can help share findings on social media for wider reach. IP15, a digital campaigner and NGO founder, suggested that women influencers can help with research dissemination.

The overall body of knowledge emerging under this theme shows that women are not passive subjects of research but are capable and have willingness to be active contributors across multiple stages of the research cycle.

### Gender and caregiving

Across both contexts, women’s involvement was constrained by patriarchal norms. Many reported needing their husbands’ permission to participate in research, and some noted the risk of gender-based violence (GBV) if they acted independently to take part. Caregiving duties further limited availability, with women often prioritising childcare, cooking, or caring for older family members over research activities. Our women proposed strategies to mitigate these barriers, including involving men in awareness activities, conducting research closer to villages to reduce travel burdens, and offering flexible scheduling and childcare support. IP15, a married woman with a son, said:

Husband’s permission is an obstacle… many women need permission to do anything. (IP15, Indonesia)

In a conversation between MP3 and MP4 (both Orang Asli and Orang Asal), both raised the risks of GBV if women go against their husbands’ wishes when participating:

Women might not want to participate because their husband does not give permission… If you oppose, they get angry and hit the wives. (MP3/MP4, Malaysia)

Across multiple LMICs, a recent systematic review shows low autonomy among married women in personal healthcare decisions, particularly in patriarchal societies where male family members dominate decision-making [9]. Such norms also restrict women’s access to phones and essential information [31] and frequently intersect with poverty [32]. A further compounding factor is women’s caregiving roles. Caregiving limits availability and underscores the need for childcare support if women from LMICs are to contribute to climate and health research. IP9, a married woman, said that women

…have a double burden, because they have to be in the domestic sphere, and they also have to be involved in the public sphere. (IP9, Indonesia)

In the domestic sphere, there is housework, homework, school runs, cooking and cleaning. In the public sphere, there is getting to work on time, working hard and earning money. This finding resonates with previous research with women in Bandung, Indonesia [33].

MP16, who advocates for gender expertise in climate justice in her job, discussed that women need childcare support:

Caregiving duties… a lot of women want to be involved, but they have children to take care of, so they need support in terms of child care. (MP16, Malaysia)

We asked participants which strategies they would find helpful, and they came up with three ideas. One was involving men, another was conducting research near villages, and the third was being flexible with their schedules.

Bringing research to the community is a strategy documented in the literature. Naserrudin et al. [34] described a study in rural Sabah, Malaysia, where participatory activities were held in community locations to build trust and ensure accessibility.

Engaging men as allies is essential to advancing women’s empowerment, as men often hold social, economic and political decision-making power within households and their communities. MP13 has previously conducted training and education with migrant workers and shared that she includes men in gender-based sexual harassment awareness training.

Ngure et al. [35] show that when men participate in gender-transformative, economic-empowerment interventions, women experience improved autonomy, mobility, and household wellbeing. Similarly, engaging men in health-promoting and GBV-prevention efforts during climate-related disasters helps to address harmful gender norms, reduce violence, and strengthen women’s positionality and agency in times of crisis.

MP6, who has not done work on gender within climate change space, would like more collaboration between climate and women’s NGOs:

So when we see climate change, we see it generally…we don’t really focus much on [gender…] We can learn from women’s NGOs, like Women’s Aid Organisation. So we know what person to ask. (MP6, Malaysia)

This theme has demonstrated the realities of caregiving and the gendered constraints that affect women’s involvement in climate and health research, with perspectives on how to overcome barriers.

### Approaches to engagement

The role of women is to find out the problems from women… the solution also comes from women. (IP16, Indonesia)

Under this theme, the women advocated bottom-up approaches that prioritise community-led problem identification and solutions, rather than top-down impositions. MP5 works with climate change-affected communities, and mentioned how the community is the core of the research and how important it is to know their expectations early:

We will ask them specifically, what are you expecting from this? … It’s so important to have the conversation of not just one party benefiting. (MP5, Malaysia)

Creative and unconventional methods, such as poetry, crafts, puppet shows, and games, were seen as effective in engaging diverse groups, including those with limited literacy. MP19 worked with an Indigenous group to raise awareness of tuberculosis (TB) and approached the matter creatively:

They had a puppet show to raise awareness… because they feel like, oh, they will understand better. (MP19, Malaysia)

MP16, a capacity-building officer, then discussed how, in the case of the Indigenous community, researchers can instead engage in crafts:

Maybe use arts and crafts… have conversations around that. (MP16, Malaysia)

The importance of accessible and multi-language dissemination was seen as a crucial marker for making information comprehensible, such as plain language, braille, and sign language, a MP3, an Orang Asli woman, who has worried that inaccessible language in research might limit Indigenous women’s full involvement, noted:

We can use simpler words… words that are easy to understand or simple for us. (MP3, Malaysia)

MP10, a disability rights activist, called for utilising Braille and sign language to ensure inclusion for the disabled community.

### Trust collaboration and leadership

The theme of Trust, Collaboration, and Leadership emerged as central to mobilising communities, fostering participation, and ensuring legitimacy in climate–health research and action. Across Indonesia and Malaysia, participants highlighted the importance of engaging chiefs, religious leaders, NGOs, and trusted intermediaries, while strengthening interdisciplinary partnerships and building trust through culturally embedded practices.

Community mobilisation was consistently described as a cornerstone for galvanising public support. In Indonesia, religious leaders were seen as particularly influential:

…the Muslim community in Indonesia predominantly listens to religious leaders. (IP8, Indonesia)

MP15, who has worked with people across intersectional groups in research, cites the importance of:

Mobilising village chiefs/community/religious leaders can help with gathering participants… involving them can be essential. (MP15, Malaysia)

These insights align with evidence from a recent systematic review, which found that religious leaders significantly shape climate–health responses by teaching environmental stewardship, collaborating with NGOs to promote ecosystem conservation, and urging congregations to protect marine environments [36]. Faith leadership thus plays a strategic role in mobilising communities towards healthier, more climate-resilient futures.

Participants emphasised the importance of interdisciplinary collaboration. Partnerships between social scientists, anthropologists, and climate-health researchers were seen as vital for bridging technical expertise and cultural context. IP3 is a social media campaigner at an environmental NGO. She noted that:

There are academics who accompany us… so actually at every step they can become collaborative partners with researchers. (IP3, Indonesia)

MP1 has research experience in local communities. She noted that climate scientists lack cultural context, and collaboration with sociologists and anthropologists can help provide an important bridge, e.g., on familiarity with traditional medicines and cultural practices.

These reflections highlight the need for knowledge exchange across disciplines, ensuring that research is both scientifically rigorous and culturally grounded.

At the heart of knowledge exchange is trust-building, and ‘trust’ emerged as a prerequisite for disclosure and participation, particularly among women in rural and marginalised communities. IP9 lives in a rural area and understands women’s reluctance to share information:

Women are not only vulnerable, but they also find it difficult to express themselves, so we need an extra approach… because it is not an easy thing. (IP9, Indonesia)

MP16, who works with women experiencing domestic violence (DV), said:

Trust is like the core or the start… They won’t tell you stories if they don’t trust you. (MP16, Malaysia)

Strategies such as food-sharing and informal conversation were described as effective ways to build rapport and reduce hierarchy. IP21 is a government health policymaker. She works on air pollution and drought-related impacts and said:

Women gather together and chat lively. (IP21, Indonesia)

In Malaysia, MP19, a medical practitioner and public health researcher, shared that she has worked closely with marginalised communities for her research and has commented on their perspectives when sharing meals:

Because they feel very connected. You eat their food, you share their food. When you eat their food, they think ‘Hey, you’re one of us.’ (MP19, Malaysia)

Research supports these practices: communal eating sustains tradition while empowering women through food decision-making, reinforcing social roles and subtly reshaping norms [37]. For researchers, such settings foster trust and document cultural change through lived experience.

Participants also emphasised the role of trusted intermediaries, including teachers, coordinators, and matriarchs, in bridging research and communities. MP19, who has had difficulties translating research into communities, discussed using teachers as facilitators as they had some understanding of research. MP6, who has previously worked with the Indigenous community, hired a local community coordinator. These figures serve as cultural brokers, translating research into community contexts and ensuring women’s voices are heard.

## Discussion

This exploratory study examined the conditions under which women in Indonesia and Malaysia might move beyond participation [1] as research subjects to become more actively involved in climate and health research. Women described clear roles they could play across the research cycle, yet their involvement was constrained by structural barriers including patriarchal norms, caregiving responsibilities, and the absence of formal mechanisms to support public involvement in research. These findings reflect broader challenges documented in LMIC contexts, where involvement and engagement remain underdeveloped compared with many high-income countries [5, 10].

The study has shed light on what is needed to create enabling environments for involvement where infrastructure for PPI is largely absent. Facilitated research platforms such as ours may provide opportunities for women to articulate their priorities, experiences, and aspirations for involvement, while also identifying the practical conditions needed to support participation.

The inclusion of women with professional research experience alongside community participants provided insight into both the opportunities and challenges of involvement. Participants identified lived experience, community networks, and cultural knowledge as important forms of expertise that can strengthen the relevance and legitimacy of climate and health research. They also highlighted trust-building, safe spaces, culturally appropriate communication, and creative engagement approaches as critical foundations for participation. These findings align with calls for greater epistemic justice in global health research [8], recognising communities [9, 12] affected by climate change as active contributors to knowledge production rather than passive sources of data [14].

At the same time, women’s accounts revealed the fragility of involvement within patriarchal contexts. Requirements for male permission, concerns about gender-based violence, restrictions on mobility, and caregiving demands all limited opportunities for participation. These findings reinforce evidence of the disproportionate burden experienced by women during climate-related events and emphasise the importance of gender-sensitive approaches to involvement [14].

The findings further suggest that involvement frameworks developed in high-income contexts cannot be assumed to transfer directly into LMIC settings [5, 10]. Rather, involvement approaches must be adapted to local social, cultural, religious, and political realities. Participants’ emphasis on community-led priorities, multilingual communication, trusted intermediaries, and culturally embedded approaches demonstrates the value of context-sensitive models for advancing involvement in climate and health research.

### Limitations

Several limitations should be considered when interpreting these findings. First, the study involved a relatively small sample of women from Indonesia and Malaysia and therefore cannot be considered representative of all women within either country or across other LMIC settings. The findings should be understood as context-specific insights rather than universally transferable conclusions.

Second, participants were recruited through governmental, community, and civil society networks, creating the possibility of selection bias. Women willing to participate may have been more interested in climate, health, or research-related issues than the wider population. In addition, social desirability bias and the influence of dominant voices within group discussions may have shaped some responses.

Third, while the study explored women’s potential roles across the research cycle, it should not be interpreted as an example of fully co-produced or community-led research. Decision-making authority regarding study design, governance, and funding remained primarily with the academic research team. The study is therefore best understood as exploring the conditions necessary for greater involvement rather than demonstrating full partnership.

Future movement towards deeper partnership would require structural changes beyond the scope of this study, including earlier involvement in agenda-setting, sustained community governance roles, investment in research literacy and leadership, childcare support, reimbursement for time, and strategies to reduce gender-based risks associated with participation and visibility.

## Conclusions

This study provides insight into how women in Indonesia and Malaysia perceive their potential roles in climate and health research, the barriers they encounter, and the conditions necessary to support more meaningful involvement. The findings demonstrate that women possess valuable expertise derived from lived experience, community networks, and cultural knowledge, but that meaningful involvement is shaped by wider structural inequalities including patriarchal norms, caregiving responsibilities, and limited institutional support.

The study highlights the importance of trust-building, culturally responsive engagement, inclusive communication, and safe participation spaces. It also demonstrates that involvement approaches must be adapted to local contexts rather than transferred uncritically from high-income settings. For researchers, policymakers, and funders, this requires investment in sustainable structures that support meaningful involvement and recognise women as contributors to knowledge production.

Ultimately, supporting women’s involvement in climate and health research is not simply a matter of inclusion. It is critical for improving the relevance, legitimacy, and sustainability of research and policy responses to climate-related health challenges. By documenting both the opportunities and barriers to involvement, this study contributes to ongoing efforts to strengthen epistemic justice and more equitable approaches to knowledge production in LMIC settings.

## Supplementary Information

Below is the link to the electronic supplementary material.

Supplementary Material 1

Supplementary Material 2

Supplementary Material 3

Supplementary Material 4

## Data Availability

Availability of data and materials for sharing is not publicly available as participants had only granted permission to the team who they had built relationships with for the purpose of the current study.

## Abbreviations

GBV: Gender based violence
HIC: High-income countries
LMIC: Low and middle-income countries
NGO: Non-governmental organizations
PPI: Patient and public involvement
