# Supplementary material for: Women’s involvement and engagement in climate and health research in two Global South countries - Indonesia and Malaysia: barriers and facilitators
Source: Res Involv Engagem. 2026 Sep 21;12:150. doi: 10.1186/s40900-026-00953-x (PMC13595536; doi:10.1186/s40900-026-00953-x)
Supplement: Supplementary file 1 [file 40900_2026_953_MOESM1_ESM.pdf]

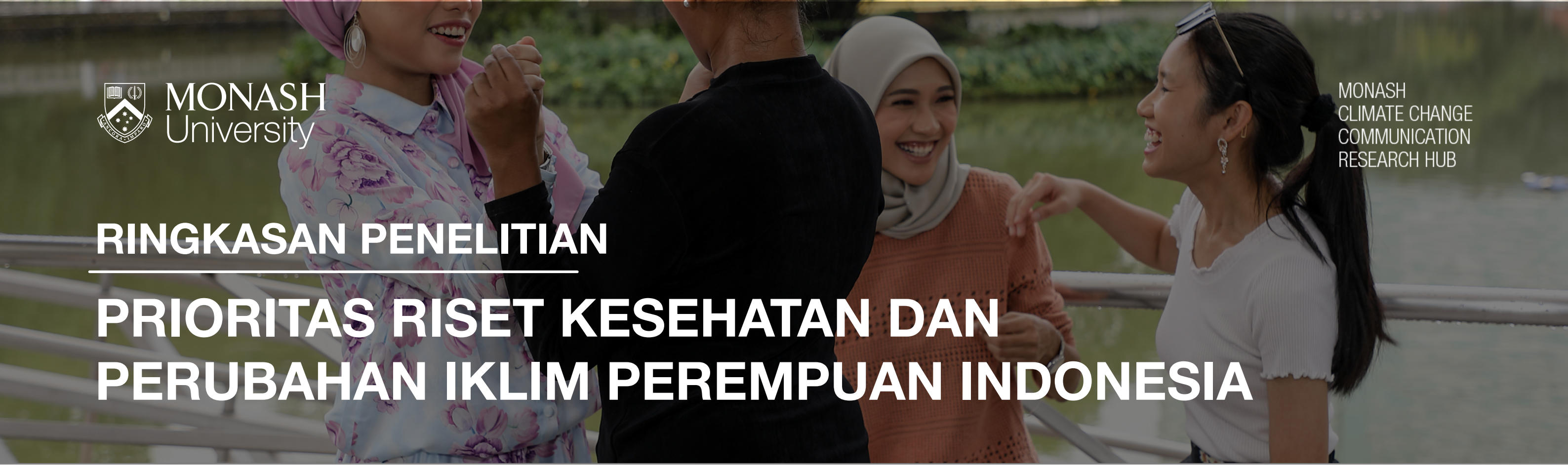

MONASH University

MONASH CLIMATE CHANGE COMMUNICATION RESEARCH HUB

RINGKASAN PENELITIAN

PRIORITAS RISET KESEHATAN DAN PERUBAHAN IKLIM PEREMPUAN INDONESIA

MENGAPA RISET INI PENTING

Perubahan iklim memiliki beragam dampak terhadap perempuan, terutama di negara berpenghasilan rendah dan menengah seperti Indonesia. Perempuan seringkali memiliki akses lebih terbatas terhadap sumber daya penting dan kerap dikesampingkan dari pengambilan keputusan dalam penetapan kebijakan riset terkait perubahan iklim dan kesehatan. Riset ini bertujuan untuk menegaskan prioritas riset perubahan iklim dan kesehatan perempuan Indonesia melalui suara mereka sendiri.

PROSES PENELITIAN

Penelitian ini merupakan bagian dari proyek yang didanai oleh Organisasi Kesehatan Dunia (WHO) untuk menyelidiki isu terkait etika yang memengaruhi penelitian perubahan iklim. Penelitian ini didanai untuk memahami prioritas penelitian iklim dan kesehatan perempuan. Dokumen ini merangkum 21 dari 24 pandangan perempuan yang diceritakan pada lokakarya yang digelar selama dua hari di Jakarta. Para perempuan direkrut melalui Lembaga Swadaya Masyarakat (LSM) yang mendukung perempuan di bidang perubahan iklim, kesehatan, dan kesejahteraan.

Lokakarya ini melibatkan perempuan Indonesia dari beragam latar belakang, mulai dari ibu rumah tangga, aparatur sipil negara (ASN), karyawan swasta, hingga aktivis yang tinggal di wilayah perkotaan maupun pinggiran kota. Usia partisipan berkisar antara 20 hingga 50 tahun.

Pada hari pertama, partisipan berbagi pengalaman pribadi dan kekhawatiran mereka tentang perubahan iklim. Pada hari kedua, para perempuan memilih tiga isu utama terkait perubahan iklim dan kesehatan menggunakan diagram dari Pusat pengendalian penyakit – *Centres for Disease Control (CDC)*. CDC adalah organisasi layanan berbasis sains dan data terkemuka di Amerika Serikat yang berfokus pada isu kesehatan masyarakat. Lokakarya diakhiri dengan perumusan rekomendasi tentang bagaimana perempuan dapat terlibat maksimal dalam penelitian.

PRIORITAS PENELITIAN PEREMPUAN INDONESIA TERKAIT PERUBAHAN IKLIM DAN KESEHATAN

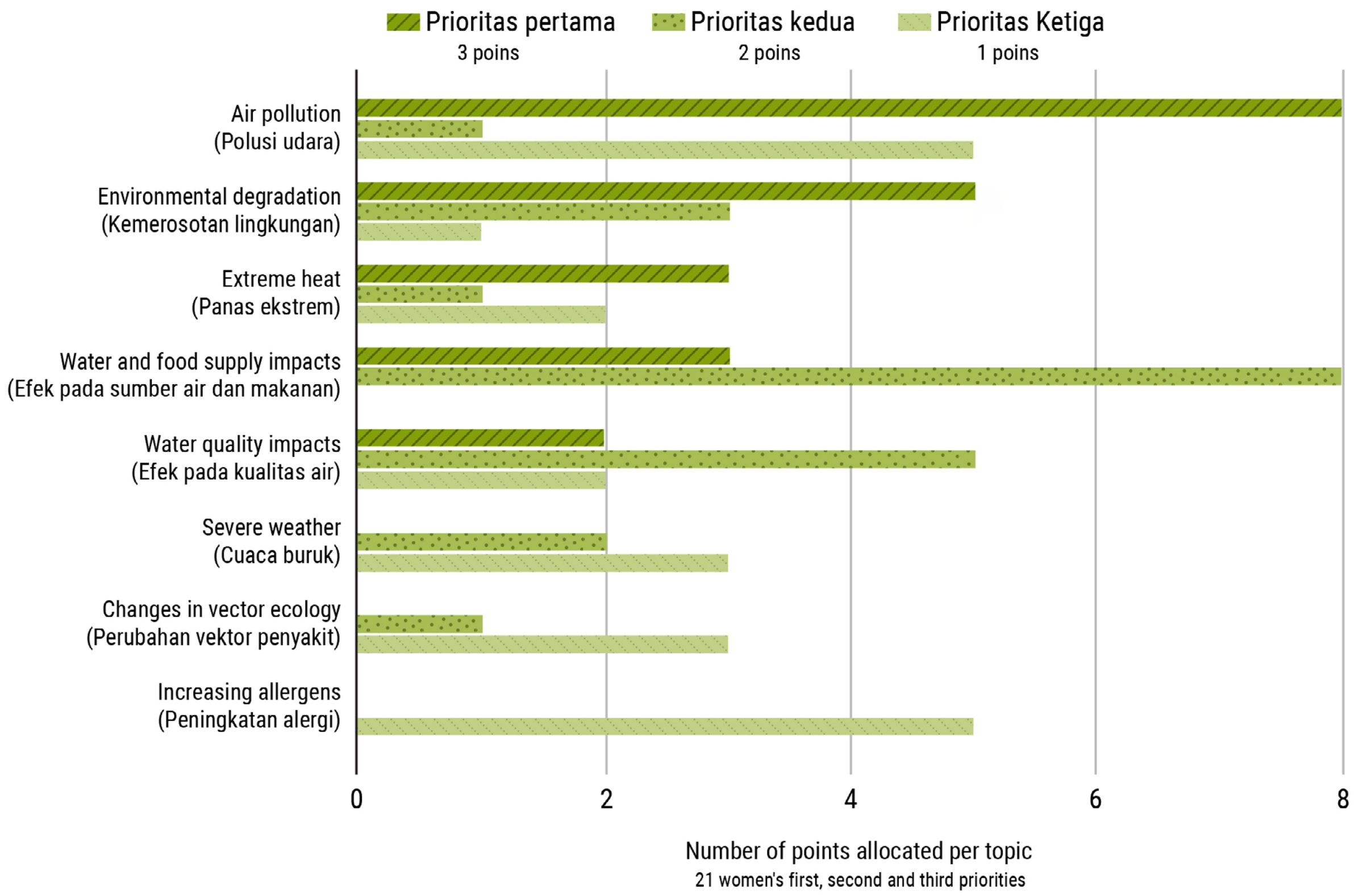

Gambar 1. Prioritas penelitian perempuan Indonesia terkait perubahan iklim dan kesehatan

TEMUAN PENTING

Temuan penting dari riset ini menunjukkan bahwa perempuan memprioritaskan polusi udara sebagai dampak kesehatan utama dari perubahan iklim. Kekhawatiran ini berakar dari realitas keseharian mereka, di mana mayoritas partisipan bekerja di pusat kota dan bermukim di kawasan penyangga Jakarta, Ibu Kota Indonesia, yang terus terpapar polusi udara. Lokasi tempat tinggal dan tempat bekerja terbukti menentukan prioritas para perempuan, topik yang kerap muncul selama lokakarya.

Sementara itu, peletakan prioritas pada isu ketahanan air dan pangan menyoroti keterkaitan erat prioritas dengan pemenuhan kebutuhan dasar. Ini merupakan tantangan yang dihadapi perempuan yang umumnya menjadi pengelola rumah tangga.

Adapun kerusakan lingkungan menjadi prioritas ketiga. Isu ini diidentifikasi sebagai pemicu dasar dari berbagai dampak kesehatan karena perubahan iklim. Partisipan mengaitkan deforestasi, pengelolaan limbah yang buruk, dan hilangnya ruang terbuka hijau dengan peningkatan suhu, penurunan kualitas udara dan air, hingga bertambahnya tekanan psikologis.

PRIORITAS 1. POLUSI UDARA

Polusi udara merupakan prioritas tertinggi. Pilihan ini merefleksikan tantangan harian sekaligus risiko kesehatan jangka panjang yang dihadapi masyarakat di perkotaan, khususnya Jakarta sebagai salah satu kota dengan tingkat polusi tertinggi di dunia.

Partisipan menyoroti polusi yang tak terhindarkan, baik di luar maupun di dalam ruangan. Hal ini tercermin dari fenomena maraknya kasus penyakit pernapasan yang menyerang anak-anak, lanjut usia, serta pekerja luar ruangan yang minim perlindungan.

> *"Polusi udara dirasakan oleh semua orang. Kasus pasien batuk terus ada sepanjang tahun." (IP2)*

> *"Polusi di Jakarta sangat terasa, terlihat, bahkan bisa diraba. Saat pandemi, polusi udara di Jakarta membaik." (IP8)*

PRIORITAS 2. DARURAT AIR DAN PANGAN

Isu ketahanan air dan pangan menempati urutan kedua. Masalah ini dipicu oleh sejumlah faktor, seperti curah hujan yang tidak menentu, kekeringan berkepanjangan, pencemaran sumber air, hingga ketergantungan pada produk pangan impor.

Para perempuan menekankan beratnya beban harian untuk memastikan ketersediaan air bersih dan pangan yang terjangkau bagi keluarga. Tantangan ini mereka kaitkan langsung dengan kesulitan ekonomi dan risiko gizi buruk (malnutrisi) pada anak mereka.

> *"Kualitas air menurun, semakin kotor dan hitam, yang berpengaruh pada penurunan kualitas pangan, seperti beras…" (IP9)*

> *"Makanan dan air adalah prioritas utama, baru kesehatan, karena sulit mencari uang dan banyak orang bahkan berhutang hanya untuk memenuhi kebutuhan (pangan dasar)." (IP4)*

PRIORITAS 3. KERUSAKAN LINGKUNGAN

Kerusakan lingkungan, yang disebabkan oleh deforestasi, limbah industri, dan aktivitas pertambangan, diidentifikasi sebagai akar penyebab berbagai risiko kesehatan. Dampak kesehatan bahkan bisa meluas, mulai dari penyakit pernapasan dan gangguan hormon hingga stres, depresi, dan kekerasan berbasis gender.

Meskipun awalnya isu ini terasa jauh bagi perempuan perkotaan, testimoni partisipan membuktikan bahwa kerusakan ekologis dianggap sebagai masalah personal karena berpengaruh terhadap ketersediaan pangan, air, dan kesehatan mental. Dampak terberat dirasakan oleh perempuan adat dan kelompok marjinal yang kelangsungan hidupnya sangat bergantung pada sumber daya alam.

> *"…Bagi kami masyarakat adat, jika alam rusak, kami kehilangan sumber pangan, kehidupan, obat-obatan, dan sebagainya." (IP22)*

> *"…di Halmahera, akses air bersih sangat terbatas akibat tambang nikel. [Setelah ada yang meninggal] Dalam satu kasus, jenazah seorang perempuan ditemukan mengandung kadar nikel di atas batas aman." (IP3)*

PRIORITAS 4. KUALITAS AIR DAN PANAS EKSTREM

Kualitas air dan panas ekstrem dinilai sebagai isu mendesak yang mulai dirasakan dampaknya. Partisipan menyatakan timbulnya berbagai penyakit akibat air minum yang tercemar, serta dampak suhu panas yang merugikan anak-anak, lanjut usia, pekerja luar ruangan, hingga hewan peliharaan.

Pengalaman peserta juga mengungkap adanya kesenjangan akses air bersih antar wilayah, mulai dari pasokan air perkotaan yang tercemar hingga kelangkaan air yang dihadapi pulau-pulau terluar. Suhu panas yang meningkat juga dirasakan telah mengganggu aktivitas harian, proses belajar, dan mengurus keluarga.

> “Banyak pulau kecil kesulitan mengakses air bersih. Beberapa bahkan sampai menampung embun. Masalah lainnya adalah limbah yang dibuang ke laut—contohnya limbah tangki septik. Bahkan di Jakarta, airnya terkontaminasi virus E. coli, tapi kebanyakan orang tidak sadar.” (IP1)

> *“Dibandingkan 20 tahun lalu, sekarang jauh lebih panas. Panasnya terasa menusuk kepala. Saya baru berani membawa anak saya keluar setelah usianya 3 tahun. Anak saya pernah mimisan karena kepanasan. Setelah itu, saya tidak lagi membawanya keluar. Anak kedua saya mengalami pusing kalau keluar rumah. Anak pertama saya bahkan sampai ada flek di paru-paru. Jadi, kalau di luar panas sekali, kami memilih di dalam rumah saja.” (IP19)*

PRIORITAS 5. PENYAKIT MENULAR

Meskipun diletakkan pada prioritas bawah, para perempuan mengidentifikasi pergeseran ekologi vektor dan bertambahnya alergen sebagai risiko kesehatan yang makin nyata berhubungan dengan perubahan iklim, yang ditandai dengan peningkatan kasus demam berdarah, malaria dan reaksi alergi. Khususnya bagi para ibu, ancaman kesehatan yang tidak terlihat ini menambah beban pengasuhan karena anak-anak semakin rentan terhadap penyakit menular.

> *“…penyakit tular vektor: anak-anak terpapar demam berdarah, malaria, dan lain-lain, yang akhirnya membuat ibu harus merawat anak [ketika sakit].” (IP14)*

REKOMENDASI

Enam rekomendasi yang dirumuskan dari temuan riset:

1. Memperbanyak riset yang berorientasi pada solusi untuk mengatasi beban domestik dan beban sosial perempuan. Hal ini bertujuan untuk mendorong keterlibatan perempuan dalam inisiatif kesehatan dan perubahan iklim di Indonesia.
2. Memetakan dampak perubahan iklim dan kesehatan berdasarkan lokasi geografis Indonesia yang beragam, sekaligus berbagai lapisan identitas perempuan.
3. Menjadikan suara perempuan sebagai fondasi dalam rancangan kebijakan perubahan iklim dan kesehatan, bukan sebatas pelengkap.
4. Meningkatkan riset partisipatif yang mengutamakan pengalaman dan kearifan lokal perempuan, termasuk praktik adat dan keagamaan.
5. Menciptakan ruang yang aman dalam seluruh proses riset agar perempuan dapat menyampaikan pandangannya secara terbuka, tanpa khawatir dihakimi.
6. Memperkuat kolaborasi antara lembaga swadaya masyarakat (LSM), peneliti, dan perempuan di komunitas akar rumput untuk mendorong pertukaran pengetahuan dan sumber daya.

LANGKAH SELANJUTNYA

Para peneliti berencana menyebarluaskan temuan ini kepada media, LSM, serta para pemangku kepentingan terkait. Para perempuan peserta lokakarya bersama tim peneliti juga akan meneruskan hasil penelitian melalui jejaring masing-masing. Selain itu, penelitian ini akan menerbitkan dua publikasi akademik.

TIM PENELITI

Seluruh tim peneliti berterima kasih kepada seluruh perempuan yang telah memberi masukan dalam mengembangkan riset bersama tim peneliti.

Dr Raksha Pandya-Wood, Western Sydney University

Dessy Rosalina, Monash University Indonesia

Dr Gabriela Fernando, Monash University Indonesia

Dr Grace Wangge, Monash University Indonesia

Savyna Selvarajan, Monash University Malaysia

Dr Azliyana Azhari, Monash University Malaysia

Nadira Reza Chairani, Monash University Indonesia

REFERENCES

US Centre for Disease Control (2024). Preparing for the Regional Health Impacts of Climate Change in the United States, p. 3

B40 is an income classification used by the Department of Statistics in Malaysia (DOSM) to categorise households based on their earnings as the bottom 40% of households in Malaysia, with income below RM5,249 per month. This group includes low-income earners who may rely on government subsidies and assistance programs.

US Centre for Disease Control (2024). Heat and Medications: Guidelines for Clinicians, p. 3-4

Penelitian ini didanai oleh World Health Organization (WHO) sebagai bagian dari Inisiatif WHO Climate change, health, ethics, yang didanai dari Wellcome Grant nomor 306679/Z/23/Z. Penelitian ini mendapatkan persetujuan etik dari Universitas Monash ID:45509; Badan Riset dan Inovasi Nasional Indonesia (BRIN) No: 084/ KE.01/SK/02/2025; dan Organisasi Kesehatan Dunia ID: ERC.0004217.
