# Supplementary material for: Women’s involvement and engagement in climate and health research in two Global South countries - Indonesia and Malaysia: barriers and facilitators
Source: Res Involv Engagem. 2026 Sep 21;12:150. doi: 10.1186/s40900-026-00953-x (PMC13595536; doi:10.1186/s40900-026-00953-x)
Supplement: Supplementary file 2 [file 40900_2026_953_MOESM2_ESM.pdf]

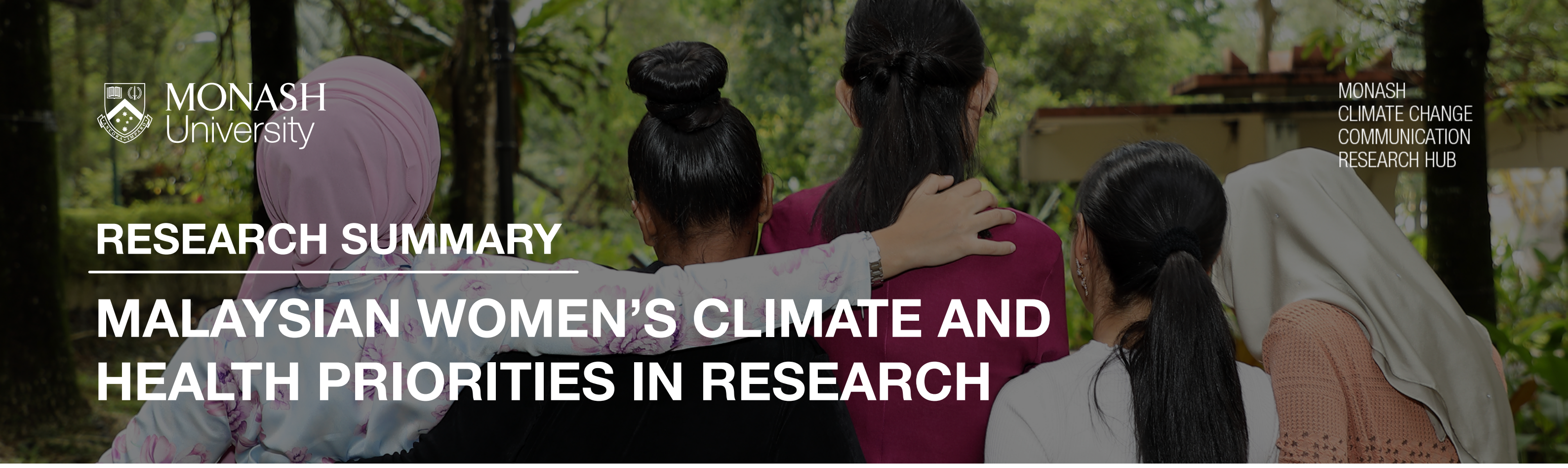

MONASH University

MONASH CLIMATE CHANGE COMMUNICATION RESEARCH HUB

RESEARCH SUMMARY

MALAYSIAN WOMEN'S CLIMATE AND HEALTH PRIORITIES IN RESEARCH

WHY THIS RESEARCH MATTERS

Climate change affects women differently, especially in low- and middle-income countries especially in Southeast Asia. Women typically have less access to resources and are often excluded from decision-making, impacting their daily lives. Current policies usually ignore gender. Women often take on caregiving roles, and due to recent climate changes, it is evident that these impacts affect not only women but also those they care for.

This study provided Malaysian women with a platform to share their experiences and to establish research priorities at the intersection of climate change and health as these issues affect them and their communities. The women co-designed this summary with the research team.

THE PROCESS

This research was part of a larger project funded by the World Health Organisation (WHO) to investigate the ethical issues affecting climate change research. This research was funded to understand women's climate and health research priorities. This document summarises 14 women's votes (out of 18, four did not vote) shared at a workshop held over two days at Monash University Malaysia. Women were recruited via organisations that support women at the intersection of climate change and health and well being. The workshop was attended by women from diverse backgrounds, including mothers, Indigenous Orang Asli (Peninsular Malaysia) and Orang Asal (Sabah and Sarawak), women with disabilities, and those from rural and urban areas.

On day one, workshop participants shared their personal experiences of climate change and on day two, they voted on their top three climate and health concerns using the US Centres for Disease Control chart[1].

KEY PRIORITIES WOMEN IDENTIFIED

Eight issues that were voted as key priorities, illustrated in Figure 1.

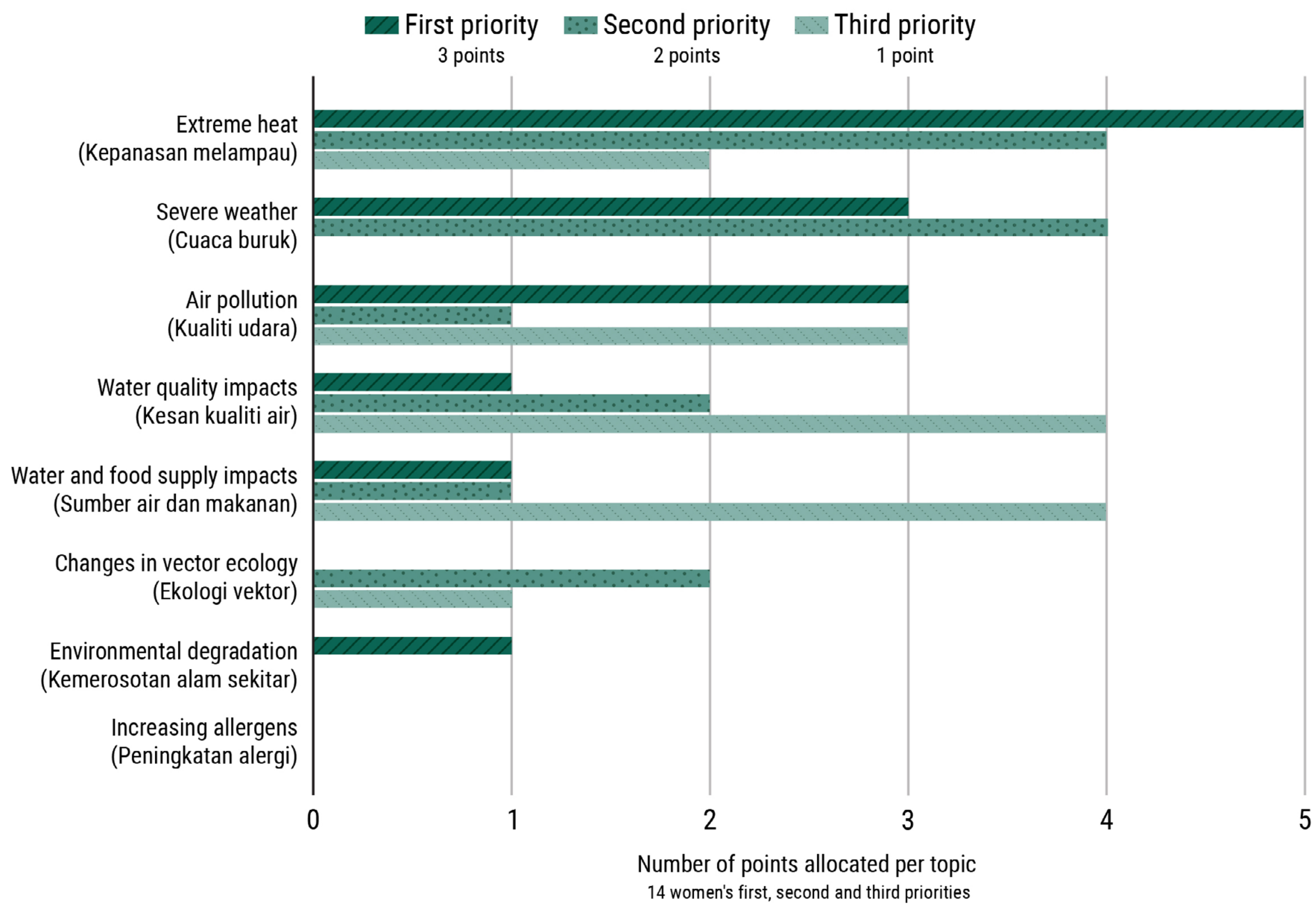

Figure 1. Chart to show women's priorities

PRIORITY 1. EXTREME HEAT

Extreme heat was the biggest concern. Women spoke about health impacts on outdoor workers, pregnant women and the elderly. They described hormonal imbalances that made it harder for them to adapt to extreme heat.

> *“Women having perimenopause or menopause; how do they adapt to extreme heat with hot flushes, mental health and physical changes? Hormonal imbalance [gets us] more irritated… mothers have anxiety when kids [are in] school under the hot weather.” (MP8)*

Another participant discussed how the Bottom 40% (B40) community[2] cannot afford to take for granted items like air conditioners to cope with high temperatures.

> *“...having access to certain things like clean water, fridges, etc. can mitigate… e.g. air conditioners to help with the heat. There is no focus on how the underprivileged are coping with this.” (MP6)*

Extreme heat led to increasing health risks among the Orang Asli. Community members reported more frequent cases of dizziness, fainting, and even heatstroke, especially among the elderly and children. These climate-related health issues add another layer of vulnerability to communities already struggling with access to clean water and food insecurity.

For women with disabilities or chronic illnesses, medications and heat interact in three primary ways:

- Thermoregulation.
- Heat can degrade or damage some medications, and patients can be counselled on how to best store their medications.
- Some medications can increase skin sensitivity from sun exposure, and patients can be counselled on skin protection[3].

PRIORITY 2. SEVERE WEATHER (STORMS)

Whilst severe weather events may overlap with extreme heat, participants expressed concerns about (severe) flooding, storms, and unpredictable rainfall patterns affecting crops. As women are often caregivers, one participant singled out how it affects their well-being:

> *“Increased rain, areas prone to flooding can create anxiety to residents, including financial consequences of flooding, issues around clean water supply. For women, this poses more issues, e.g., for women menstruating or with children.” (MP11)*

Another mentioned that she would have to deal with spoiled food from house blackouts, and raised how women would be exposed to toxic waste from floods, and would thus fall ill:

> *“My house, if it thunders, it will trip the electricity, and I think about the food in the fridge will get ruined. If it’s too loud or frequent [thunder], it will trip [ruining] my leftovers in the fridge - its economic impact as well… Flash flood in the city - when it rains so heavily, roads get blocked… Women clean up in the house after the flood. [potentially exposing themselves to] Toxic pollution.” (MP1)*

The rainy season also leads to job losses for rubber tappers within the Orang Asli community, as this is their main source of income. From the food source aspect, many crops fail to grow well, and it is difficult to catch fish because the water is murky due to flooding. This can then endanger their livelihoods.

PRIORITY 3. AIR POLLUTION AND BREATHING DIFFICULTIES

Air pollution was a key concern among the participants. Urban residents pointed out motor vehicle emissions and open burning as daily irritants that affect those who have breathing difficulties, such as asthma. Women noted the link between poor air quality and the increase in respiratory conditions in children and the elderly. The following quote summarises this sentiment:

> *“Air pollution... burdens women [labourers] as they have to take care of their sick kids.” (MP17)*

Another participant raised the concerns of long-term mask wearing due to air pollution, especially for outdoor workers:

> *“Air pollution requires mask wearing, and depending on occupations, say factory workers, it might lead to difficulty in breathing and heat stroke. For some plantations, each worker is only given one mask to use for a long time.” (MP13)*

PRIORITY 4. WATER QUALITY AND FOOD SUPPLY CHALLENGES SPECIFIC TO ORANG ASLI AND ORANG ASAL

There was a clear difference between the Orang Asli and Orang Asal participants and the others, with water being a main priority. This is because the Orang Asli and Orang Asal live near rivers and heavily rely on them. These rivers also often get polluted due to illegal activities such as logging. This affects their health and livelihoods as a result:

> *"Rivers are a direct resource. Logging has polluted the rivers, causing the water to look like milky tea. My community spoke against the logging, and the loggers stopped their activities for a while, but the river remains stagnant. As a result, my community found a river source that is clean but small, and built a small dam. That said, some people still use the water from the polluted river which gives them skin diseases because the water is caked in mud." (MP12)*

In addition to skin diseases, these communities can potentially be infected by E.Coli bacteria as a result of consuming food and water from polluted rivers. With climate change also affecting rainfall patterns, these critical water and food issues are likely to be further exacerbated.

PRIORITY 5. DATA GAPS

Vector ecology (organisms that transmit disease), general environmental degradation, and increasing allergens were scored low; the women raised that this was due to a lack of Malaysian data around these issues:

> *"There were some priorities like "increasing allergens" and "vector ecology changes" that I hesitated to rank highly due to a lack of localised data or community awareness, rather than lack of importance." (MP18)*

This highlights the need for further awareness and research on emerging or less visible health issues.

In addition, one participant pointed out that forced migration should be included as a priority not reflected in the CDC categories. There is a growing number of refugees in the country; often facing physical and mental challenges that affect their health:

> *"There's very little on forced migration. Nobody acknowledges forced migration in Malaysia… Especially on mental health…" (MP9)*

RECOMMENDATIONS

Based on these priorities, we have seven recommendations going forward:

1. Proactively include diverse women's voices in climate and health policymaking from the start, mandating it at every stage of policymaking.
2. Invest in gender-responsive health systems that also integrate traditional ecological knowledge systems and culturally grounded health indicators.
3. Support participatory research that values and reflects women's lived experience and knowledge, and builds their research capacity.
4. Create safe spaces in research processes against their other responsibilities, so that women can share openly without judgment and concerns for their other duties.
5. Recognise women as care partners and include their perspectives in policies.
6. Amplify women's leadership in health governance bodies, climate policy committees, and research design teams.
7. Strengthen collaboration between NGOs, researchers, and communities to share experience, knowledge, and resources.

NEXT STEPS

Researchers will share the work with the media, NGOs, and other stakeholders with whom they are connected. The women who participated in the workshop and the research team will share this briefing with their connections. Two academic publications are also being developed.

AUTHORS

We wish to thank the women for their comments and help for co-developing this summary with the research team.

Dr Raksha Pandya-Wood, Western Sydney University

Dessy Rosalina, Monash University Indonesia

Dr Gabriela Fernando, Monash University Indonesia

Dr Grace Wangge, Monash University Indonesia

Savyna Selvarajan, Monash University Malaysia

Dr Azliyana Azhari, Monash University Malaysia

Nadira Reza Chairani, Monash University Indonesia

REFERENCES

1 US Centre for Disease Control (2024). Preparing for the Regional Health Impacts of Climate Change in the United States, p. 3

2 B40 is an income classification used by the Department of Statistics in Malaysia (DOSM) to categorise households based on their earnings as the bottom 40% of households in Malaysia, with income below RM5,249 per month. This group includes low-income earners who may rely on government subsidies and assistance programs.

3 US Centre for Disease Control (2024). Heat and Medications: Guidelines for Clinicians, p. 3-4

This research was funded by the World Health Organisation as part of the WHO climate change, health, ethics initiative which has been supported by Wellcome Grant 306679/Z/23/Z. The study was granted ethical approval by Monash University ID:45509; National Research and Innovation Agency Indonesia (Badan Riset dan Inovasi-BRIN) No: 084/ KE.01/SK/02/2025; and World Health Organization ID: ERC.0004217.
