# Supplementary material for: Women’s involvement and engagement in climate and health research in two Global South countries - Indonesia and Malaysia: barriers and facilitators
Source: Res Involv Engagem. 2026 Sep 21;12:150. doi: 10.1186/s40900-026-00953-x (PMC13595536; doi:10.1186/s40900-026-00953-x)
Supplement: Supplementary file 3 [file 40900_2026_953_MOESM3_ESM.docx]

Appendix 3: Participants anonymised profiles: Indonesia and Malaysia

| Alias name | Profile |
| --- | --- |
| MP1 | a Fijian Indian woman based in urban Asia, leverages her positional privilege to center the voices of climate-affected communities across the Asia-Pacific. |
| MP2 | A translator who breaks the barriers faced by Orang Asli women by bridging the digital and language divide. |
| MP3 | An Orang Asli, mother of three and first-time worker, balances foraging, cooking, and gibbon care within a low-wage conservation role. |
| MP4 | Young Orang Asal media student who face digital and language exclusion but play key roles in knowledge mediation and cultural continuity |
| MP5 | A Gen Z Chinese zero-waste campaigner with a legal background, actively engages with marginalized communities affected by climate change |
| MP6 | A campaigner who struggles with anxiety. Prioritises community engagement and translation but lacks gender-climate lens. |
| MP7 | Not directly impacted by climate events but works with indigenous community, fffers logistical and research support |
| MP8 | Works as health team members with deep experience with disaster-impacted communities (refugees, migrants, stateless) |
| MP9 | Works as health team members with deep experience with disaster-impacted communities worked with marginalised communities impacted by climate-related disasters, such as providing menstrual kits, SRH assistance, etc. |
| MP10 | A disabled Malay-Muslim woman and advocate from Kuala Lumpur, experiences direct health impacts from air pollution and heat, such as blocked airways. |
| MP11 | A Malay-Muslim woman with thyroid issues and allergies aggravated by heat and pollution, is also a primary caregiver to her disabled sibling |
| MP12 | Lives near a highway, has urban mobility, but committed to sharing lived experiences of Indigenous women. |
| MP13 | She works as at migrant rights organization with focus on rights education and mutual learning models. |
| MP14 | She works as programme officer at women rights community and lives with sinus issues. |
| MP15 | Indian-Muslim, respiratory issues from air pollution with experiences in research. |
| MP16 | Capacity officer at allergy and clinical immunology societies and focus on gender expertise in climate justice. |
| MP17 | She works at allergy and clinical immunology societies and wants caregiving roles centered in climate-health narratives. |
| IP1 | She handles household bills—a role uncommon for women in Indonesia and eco-consciousness amidst Jakarta's urban climate risks |
| IP2 | A doctor and health NGO worker who teaches first aid in climate-affected communities. |
| IP3 | She works as Gen Z social media campaigner for environmental NGO and affected by COVID-19 anxiety. |
| IP4 | She is ojol (motorcycle taxi) driver and union representative with mental health issues and lacks health insurance. |
| IP5 | A midwife, lecturer, and doctoral student who runs a warteg (food stall), donates unsold food. |
| IP6 | She lives in the industrial zone, where pollution and climate stressors intersect with her work in health policy research. |
| IP7 | A government health officer who manages climate-related planning in public institutions. |
| IP8 | She works for environment NGO & religious community and focus to give her a unique perspective on integrating religious values into climate and health narratives. |
| IP9 | She lives in rural area and works to empower women-headed households through sustainable supply chain initiatives. |
| IP10 | A young women’s advocate who works on early marriage and empowerment through women national commision. |
| IP11 | A plastic reduction advocate who affected by allergies due to climate change. |
| IP12 | A nurse and public health master's student who also an activist for chronic health community. |
| IP13 | A health NGO worker who lives with allergies and mental health challenges, which are worsened by climate-related factors. |
| IP14 | A researcher and part of the sandwich generation who experience in climate-related research on waste and food. |
| IP15 | She works as a digital campaigner turned NGO founder who wants a safer climate for her son. |
| IP16 | An environmental journalist living with bipolar disorder, manages her health through medication, exercise, and strict diet while also being a caregiver. |
| IP17 | She works as a health NGO worker, mother of two. Nearing menopause, advocates for elderly and women's health. |
| IP18 | A motorcycle driver and activist with online driver organization who confronts daily exposure to extreme weather. |
| IP19 | Health worker NGO leader and active on urban poor communities. |
| IP20 | Journalist from environmental and religious community in Indonesia who navigates extreme heat (cats suffer mouth ulcers) and prefers digital engagement. |
| IP21 | Government health policymaker who works on air pollution and drought-related impacts. |
| IP22 | Dayak weaver and planetary health advocate who balances intergenerational expectations with global youth identity and indigenous ecological knowledge. |
