# Supplementary material for: Women’s involvement and engagement in climate and health research in two Global South countries - Indonesia and Malaysia: barriers and facilitators
Source: Res Involv Engagem. 2026 Sep 21;12:150. doi: 10.1186/s40900-026-00953-x (PMC13595536; doi:10.1186/s40900-026-00953-x)
Supplement: Supplementary file 4 [file 40900_2026_953_MOESM4_ESM.docx]

Appendix 4 GRIPP 2 Checklist Women’s involvement and engagement in climate and health research in the Global South: barriers and facilitators

| Section and Topic | Item | Section where reported and details |
| --- | --- | --- |
| Aim | Report the aim of patient and public involvement (PPI) in the study. | Barriers to and facilitators of women’s (including Indigenous women’s) active involvement and engagement in climate and health research. See P3. |
| Methods | Provide a clear description of the methods used for PPI in the study. | This paper reports part two of a research study: Part 1 was about women sharing their research priorities for climate and health research and then creating summaries of these priorities for stakeholders (note - this is not the focus of the current paper - see appendix 1 and 2 for these summaries of their priorities). We discuss the wider involvement of women in the research process. Women from Indonesia and Malaysia helped the research team with data collection and analysis (NRC is also a co-author in the current article). We share women's experiences of what involvement in the research process looks like and could look like. See P4,7. |
| Study results | Outcomes  Report the results of PPI in the study, including both positive and negative outcomes. | Our work has found that women’s involvement in climate and health research cannot be assumed but must be actively and intentionally enabled through deliberate strategies such as safe spaces, childcare support, and culturally sensitive facilitation. See P23. |
| Discussion and Conclusions | Outcomes Comment on the extent to which PPI influenced the study overall. Describe positive and negative effects. | The research team itself was made up of women from Indonesia and Malaysia (two of whom are community members) who have begun to get involved in research processes. Having their local knowledge was helpful to help demystify involvement and engagement in research with the other women who attended the workshops. NRC co-authored the current paper but English is not their first language so they helped with the Bahasa Indonesian analysis and summaries mostly and the plain language summary. |
| Reflections/Critical perspective | Comment critically on the study, reflecting on the things that went well and those that did not, so others can learn. | Researchers working with local women, adopt bottom-up, creative, and multi-language and accessible approaches that respect local knowledge and build trust using the arts and engaging techniques. Researchers also need to form collaborations with religious leaders and work with men as allies. |
